# The Preparation and Characterization of *N*,*N*-Dimethyl Acrylamide-Diallyl Maleate Gel/Hydrogel in a Non-Aqueous Solution

**DOI:** 10.3390/gels9080598

**Published:** 2023-07-25

**Authors:** Md Murshed Bhuyan, Mobinul Islam, Jae-Ho Jeong

**Affiliations:** 1 Thermal-Fluid Energy Machine Lab., Department of Mechanical Engineering, Gachon University, 1342, Seongnam-daero, Sujeong-gu, Seongnam-si 13120, Republic of Korea; 2 Department of Energy and Materials Engineering, Dongguk University, Seoul 04620, Republic of Korea; mobin01cjnu@gmail.com

**Keywords:** non-aqueous, gel, gamma radiation, DMAA, DAM

## Abstract

A few drugs need non-aqueous gels for release in the specific region of the intestine. The present work focuses on preparing *N*,*N*-Dimethyl acrylamide-Diallyl Maleate (DMAA-DAM) gel in Dimethyl sulfoxide (DMSO) solvent by applying different doses of gamma radiation and then characterization. The blend solution of 10%: 10%—DMAA: DAM was prepared in DMSO and irradiated at 2, 5, 10, 20, and 30 kGy doses from the Co-60 gamma source. After extraction, it was observed that all of the radiation doses yielded more than 95% gel content. The best gel content was found for 10 kGy dose, which was 97%. The equilibrium swelling was optimized 1800% of the dried gel for 5 kGy dose. Gel formation was confirmed by analyzing characteristic functional groups and the environment of protons in the gel structure by using FTIR and NMR spectroscopy. The thermal stability was tested using DSC and TGA which showed the glass transition temperature at 86.55 °C and the degradation started at 320 °C. The XRD pattern analysis revealed the semi-crystalline nature of the gel. Therefore, DMAA-DAM gels can be a good candidate for use in different fields of study, especially in drug delivery.

## 1. Introduction

Gels are semi-solid or semi-liquid polymeric materials consisting of long-chain molecules cross-linked with one another. There are two types of gel: (i) chemical gel and (ii) physical gel [1]. If the gel can retain a large amount of water without dissolving in it, then it is called hydrogel [2]. Due to their diverse properties, gels are widely used in metal adsorption [3], agriculture [4], electronics [5], biomedical engineering [6], and drug delivery [7]. Usually, co-polymeric hydrogels are prepared in aqueous solution by applying gamma radiation. Water molecules participate directly in the polymerization mechanism of hydrogels through the formation of hydrogen and hydroxyl free radicals. The hydroxyl free radicals strike and subtract the proton from the reactant monomers to produce their free radicals. Then, the free radicals propagate and terminate the copolymerization reactions to produce the gel products [8]. But, irradiating monomers in a non-aqueous solution may not follow the same reaction mechanism which created the interest to work on ‘gamma radiation induced gels in non-aqueous media’. In many cases, non-aqueous hydrophilic and hydrophobic gels are required for the delivery of a few specific drugs [9]. Because of hydrolysis, drugs that are poorly soluble/insolubility in water are not suitable/stable in aqueous media to release in the intestine. So, non-aqueous gels are required to mitigate the situation through the preparation of different solvents rather than water. Anthony Tablet and Chun Wang studied and broadly explained the advantages, limitations, and future prospects of non-aqueous gel/supra-molecules in the field of drug delivery [10]. Yong Zhang et al. prepared non-aqueous gel via the solvent evaporation method and used it in transdermal drug delivery, where they found a steady release at an expected rate [11]. Monomers should also be eligible for the formation of gel upon gamma radiation exertion in a non-aqueous medium. DMAA is a nonionic monomer that easily undergoes all sorts of polymerization such as chemical and irradiation techniques. Its hydrogel shows amelioration in swelling in different environments, thermal and mechanical stability, and self-healing properties which promote its applicability in toxic metal adsorption, dye removal, and biomedical engineering [12,13]. Owing to their stimuli-responsive nature, DMAA gels are being used to deliver various kinds of drugs into the intestine [14]. To overcome the limitations (lower mechanical strength [15]) of DMAA gels, another monomer can be incorporated with it. DAM can be an efficient candidate for blending with DMAA in non-aqueous media. DAM is a trifunctional monomer usually used as a cross-linking and branching agent. It has a tendency to form cyclo-copolymer as the allyl double bond is less reactive than vinyl double bonds [16]. Both DMAA and DAM yield gel in non-aqueous solutions using the solvent like DMF, CCl4, DMSO, and Benzene, etc. F. Martellini et al. reported on 2-methoxyethylacrylate-*N*,*N* dimethylacrylamide hydrogel prepared by applying gamma-radiation-induced polymerization in dimethylformamide solution [17]. Kunio Urushido explained the intramolecular cyclization of diallyl maleate and broadly discussed its radical polymerization in benzene solvent at 60 °C in the presence of 2,2-azobisisobutyronitrile [18]. Therefore, DMAA and DAM may combine to produce a gel. Dimethyl sulfoxide (DMSO) is a water-soluble organic solvent generally used singly or by mixing with other solvents to dissolve solute and proceed with polymerization upon the exertion of gamma irradiation [19]. Due to the lack of use of initiators and cross-linking agents, among all of the methods, the gamma radiation technique is the most promising, effective, and prioritized for the preparation of pure gel. Gamma radiation is a highly energetic (<0.25 Å wavelength, >12 EHz (1 EHz = 10^18^ Hz) and >50 keV energy) electromagnetic ray that can initiate copolymerization in both aqueous and non-aqueous solutions [17,20]. However, a detailed study regarding DMAA-DAM gels in non-aqueous solutions was not yet reported. The aim of the current work is to prepare a polymeric gel from DMAA-DAM in DMSO solution by applying different doses of gamma-ray, optimization of radiation dose, and characterization.

## 2. Results and Discussion

### 2.1. Radiation Polymerization of DMAA-DAM Gel

**Scheme 1:**
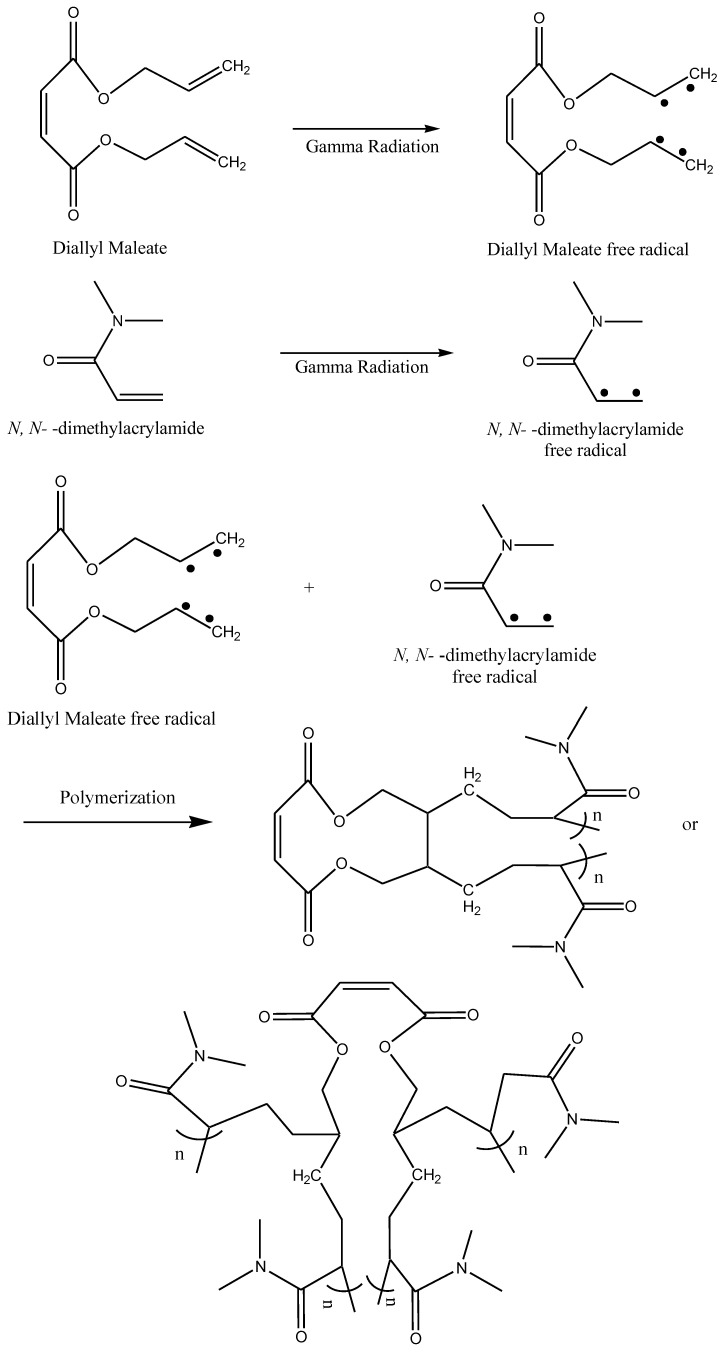
Probable polymerization mechanism of DMAA-DAM gel.

**Figure 1:**
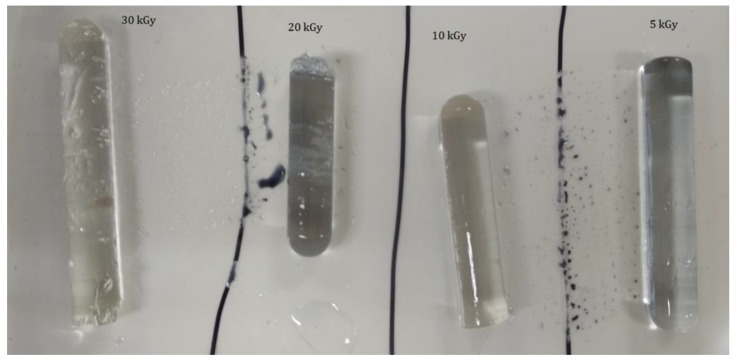
DMAA-DAM gels after preparation.

Gamma radiation polymerization was performed in a non-aqueous DMSO solution by applying radiation doses 2, 5, 10, 15, and 20 kGy, respectively (Figure 1). At the lower radiation dose (2 kGy), the hydrogel was not found, which may be attributed to insufficient activation energy to activate the monomers for starting the reaction. Generally, in the case of an aqueous solution, gamma rays split the water molecules into hydrogen and hydroxyl free radicals which initiate the reaction. Herein, the mechanism should follow a different pathway. Since the medium is non-aqueous DMSO solution, the polymerization mechanism may be attributed to the formation of free radicals upon exerting the gamma rays onto the solution of monomers. DAM free radicals may go through intra-cyclization and chain propagation with DMAA free radicals, or smaller monomer DMAA may act as a linker between DAM homopolymers [16]. Another possibility is the direct chain propagation and termination between DMAA and DAM free radicals, shown in Scheme 1. It can be concluded that gamma ray polymerization in a non-aqueous DMSO solution is a feasible and effective method of producing hydrogels with tunable properties for various applications.

### 2.2. Effect of Radiation Dose on DMAA-DAM Gel Content

**Figure 2:**
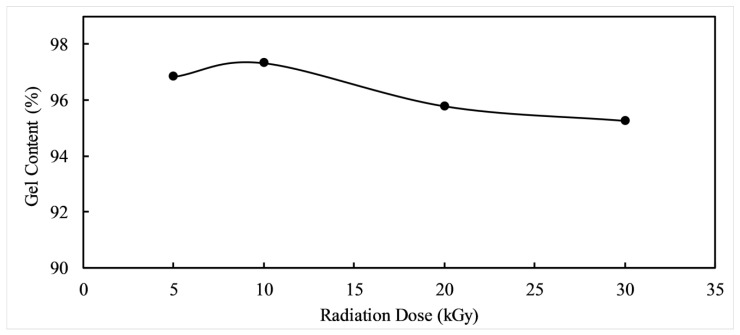
Effect of radiation dose on gel content of DMAA-DAM gel.

The gels were extracted in water to remove the water-soluble DMSO, unreacted monomers, homopolymers, and other contaminants. Figure 2 presents the gel content of the DMAA-DAM gel prepared for 5, 10, 20, and 30 kGy radiation doses. The gel content does not show much difference and lies between 95 and 97% indicating that the range of doses used here is suitable for the preparation of the gel. Yet, the small variation in gel content can be attributed to the lower radiation dose activating the maximum amount of reactors to the transition state, resulting in higher products. In contrast, the higher radiation doses may degrade a few monomers and subsequently produce a smaller amount of product. Therefore, the lower dose of 5 or 10 kGy can be optimized for the preparation of the gel [21].

### 2.3. Effect of Radiation Dose on the Hydrophilicity/Equilibrium Swelling of Gels

**Figure 3:**
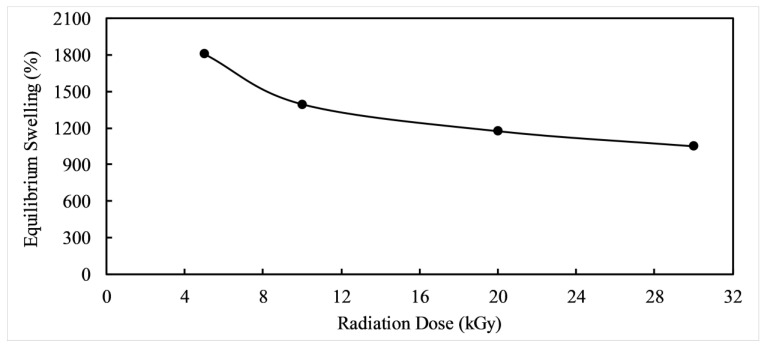
Effect of radiation dose on hydrophilicity of DMAA-DAM gel.

**Figure 4:**
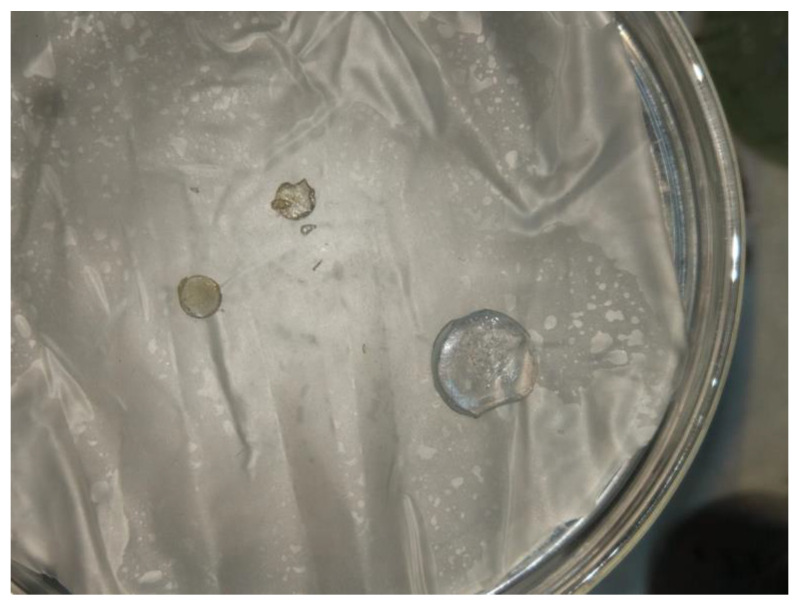
MAA-DAM gel before and after swelling prepared with 10 kGy radiation dose.

Equilibrium swelling keeps vital rules in the efficiency of drug delivery from gels. The DMAA-DAM gel possesses the functional group responsible for hydrophilicity. The equilibrium swelling was performed in a neutral medium (pH~7.0) to investigate the degree of hydrophilicity. Figure 3 depicts the effect of radiation dose on the gel network followed by a maximum amount of water absorption/adsorption (equilibrium swelling) in the void spaces. It is clear from the graph that the hydrophilicity decreases with the increasing exerted radiation dose. In the case of a lower dose, most of the monomers become involved in the gel formation with the lower cross-linking density, resulting in the larger void space that can hold large amounts of water. On the other hand, at the higher radiation dose, monomers produce denser cross-linking in the gel network which can reduces the hydrophilicity and equilibrium swelling [22]. It is noticeable that the DMAA-DAM gels show lower equilibrium swelling compared to other gels in aqueous media. The gels may undergo intramolecular hydrogen bonding which shrinks the network and disables absorption/adsorption sites [23,24]. The swelling may be improved by changing the swelling media, pH, and temperature [25]. Therefore, for higher swelling, a lower radiation dose can be optimized. Figure 4 exhibits the states of gels before and after swelling in neutral pH media, which reflects the stability after swelling.

### 2.4. Characterization of Gel Using FTIR Spectroscopy

**Figure 5:**
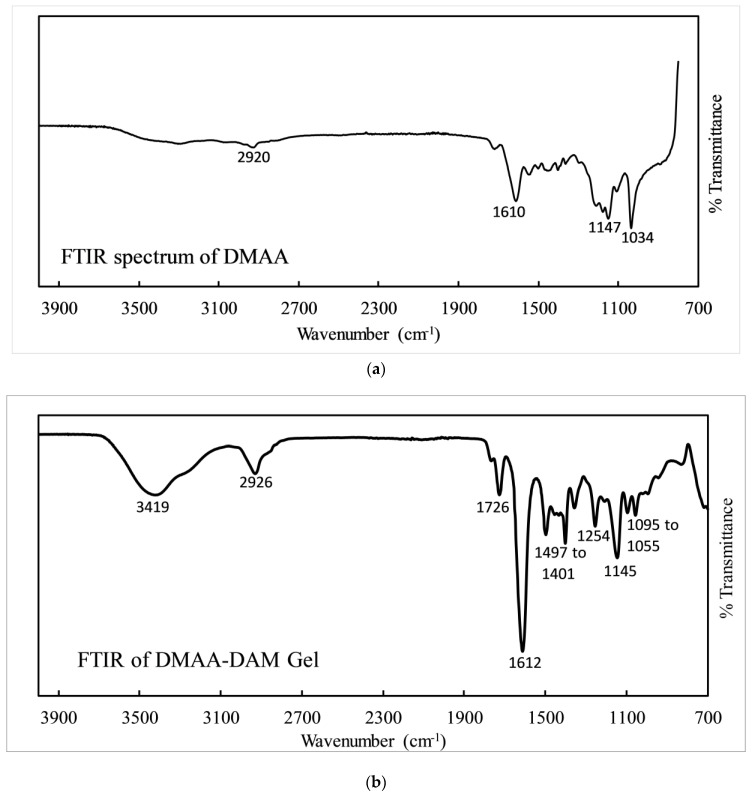
FTIR spectra of (**a**) DMAA and (**b**) DMAA-DAM gel.

Figure 5 represents the FTIR spectra of DMAA and DMAA-DAM gel. In Figure 5a the peak at 2920 cm^−1^ for the –C-H stretching of the hydrocarbon chain and at 1610 cm^−1^ for the –C=O stretching of the tertiary amide group, 1147 cm^−1^ and 1034 cm^−1^ are assigned to the –C-N stretching of amide. In Figure 5b, the FTIR spectrum of DMAA-DAM gel showed a peak at 3419 cm^−1^ for moisture present in the network. All of the characteristic peaks are present with little changes in the energy due to copolymerization. The peaks at 1726 cm^−1^ for the –C=O stretching of DAM, 1497 to 1401 cm^−1^ for the C-N stretching of gel, 1254 cm^−1^ for the asymmetric –C-O-C tensile vibration, and 1145 cm^−1^ and 1095 to 1055 cm^−1^ are assigned to –C-O-C symmetric tensile vibration [26]. Therefore, the DMAA and DAM undergo gel formation.

### 2.5. The Characterization of Gel Using Proton NMR Spectroscopy

**Figure 6:**
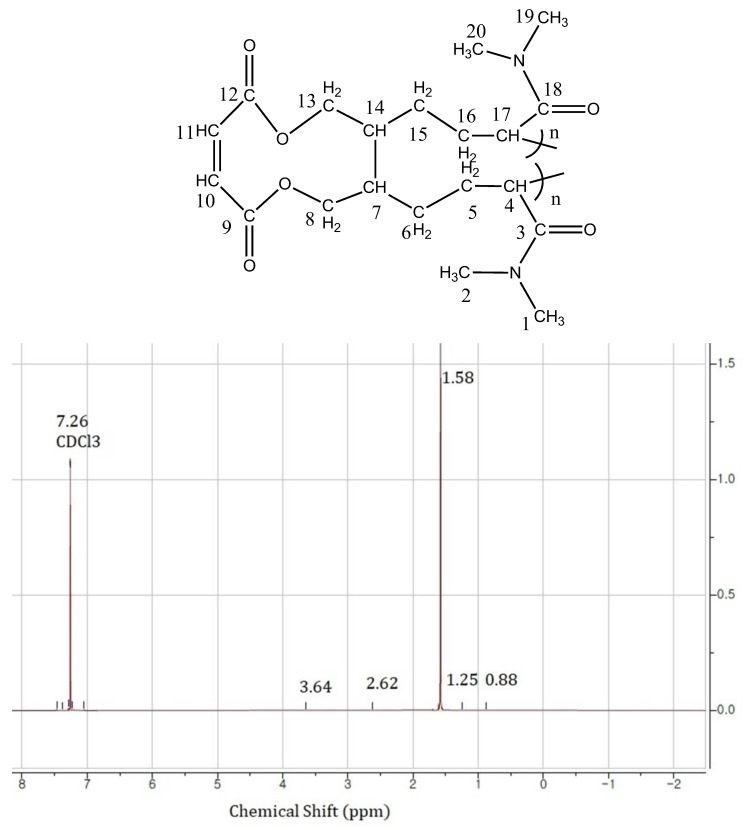
^1^H NMR peaks of DMAA-DAM gel.

Since the gel is not well miscible with chloroform solvent, the intensity of the NMR peaks is not satisfactory. In Figure 6, the peaks at 1.58 and 7.26 are for the reference and solvent. The gel shows peaks at 0.88 and 1.23 for methyl (-CH_3_-) protons (carbon no. 1, 2, 19, 20). The peak at 2.62 is for –CH- protons (carbon no. 4, 7, 10, 11, 14, 17), and the peak at 3.64 s corresponds to methylene (-CH_2_-) protons (carbon no. 5, 6, 8, 13, 15) [27].

### 2.6. Thermal Analysis Using Differential Scanning Calorimetry (DSC)

**Figure 7:**
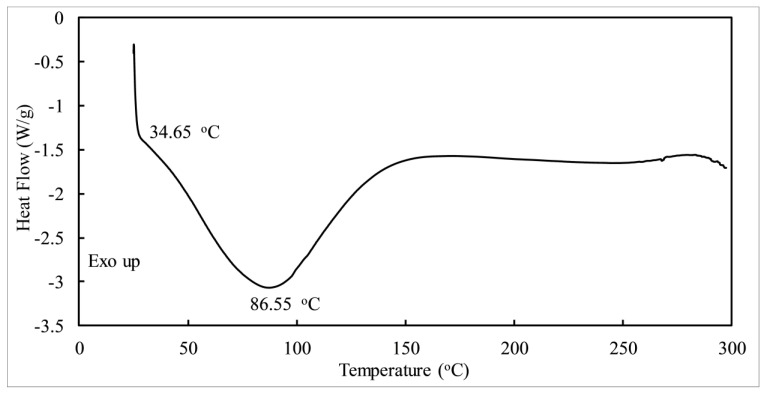
DSC thermogram of DMAA-DAM gel prepared with 10 kGy radiation dose.

Figure 7 demonstrates the graph of temperature against heat flow where the primary inclination (at 34.65 °C) is due to the escaping moisture from the gel. The glass transition temperature is at 86.55 °C, whose endpoint is around 150 °C. From the DSC curve, it can be concluded that the gel does not melt or decompose below 150 °C, which implies that the gel has good thermal stability under atmospheric conditions [28]. The melting temperature of the gel is expected to be above 150 °C, which can be further confirmed via thermogravimetric analysis (TGA).

### 2.7. Thermogravimetric Analysis (TGA)

**Figure 8:**
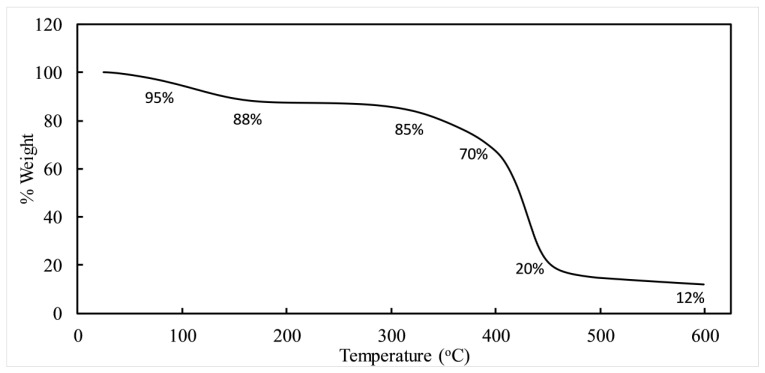
TGA graph of DMAA-DAM gel prepared with 10 kGy radiation dose.

Thermogravimetric analysis (TGA) is a technique that measures the change in the weight of a sample as it is heated or cooled over a range of temperatures or time intervals. The TGA curve shows how the weight of the sample varies with temperature or time. The TGA curve of the gel is shown in Figure 8 and can be divided into four segments that correspond to different physical and chemical processes occurring in the sample. The first segment of the TGA curve starts from 25 °C and ends at 86 °C, where the weight of the gel decreases by 5%. This segment represents the initial dehydration of the gel, which contains some water molecules that are released as vapor when heated. The second segment of the TGA curve starts from 86 °C and ends at 157 °C, where the weight of the gel decreases by another 7%. This segment corresponds to the transition of the gel from a rubbery state to a glassy state, which is also observed in the DSC graph as an endothermic peak. The glassy state is a rigid and brittle state of the gel that has low molecular mobility. The third segment of the TGA curve starts from 157 °C and ends at 320 °C, where the weight of the gel remains relatively constant at around 85%. This segment indicates that the gel is stable in its glassy state and does not undergo any significant changes in its structure or composition. The fourth segment of the TGA curve starts from 320 °C and ends at 600 °C, where the weight of the gel decreases drastically by 73%. This segment represents the main thermal degradation of the gel. The gel melts at around 320 °C and then degrades into smaller carbonaceous products that are vaporized at higher temperatures. Above 500 °C, only about 12% of the original weight of the gel remains, which mainly consists of inorganic residues that are thermally stable. The TGA curve does not show any significant changes above this temperature [28]. So, it can be concluded that the TGA graph reflects stability at normal temperature and pressure.

### 2.8. Surface Analysis with SEM-EDS

**Figure 9:**
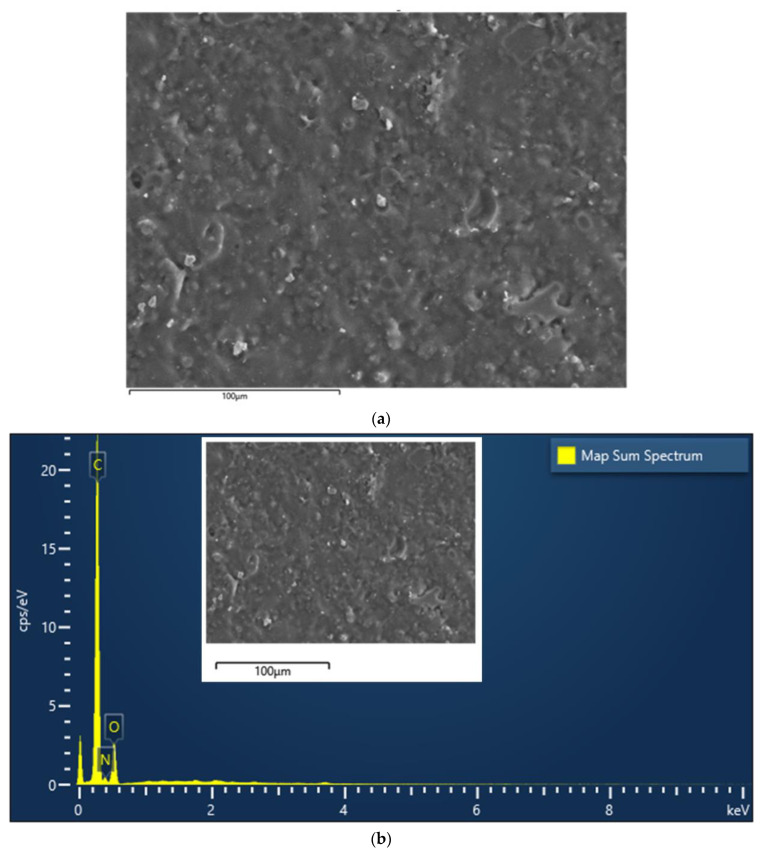
(**a**) SEM and (**b**) SEM-EDS of the DMAA-DAM gel.

Scanning electron microscopy energy dispersive spectroscopy (SEM-EDS) is a technique that combines the imaging of the surface morphology of a sample with the detection of the elemental composition of the sample [29]. The SEM-EDS analysis requires the sample to be coated with a conductive material, such as platinum, to prevent charging and improve image quality. In this study, the SEM-EDS analysis was performed on the dried DMAA-DAM gel that was prepared by irradiating the DMAA-DAM solution with a 10 kGy radiation dose. The objective of the SEM-EDS analysis was to examine the surface structure, texture, and chemistry of the gel for potential applications. The SEM image of the gel is shown in Figure 9a and reveals the rough and entangled surface of the gel. The roughness and entanglement of the gel are attributed to the cross-linking and polymerization of the DMAA-DAM molecules during irradiation, which create a three-dimensional network of interconnected channels in the gel matrix. The network of the gel is beneficial for applications that require a high surface area, water absorption, or drug delivery. The EDS spectrum of the gel is also shown in Figure 9b and displays the peaks corresponding to the elements present in the gel. The EDS spectrum confirms that the gel consists mainly of carbon (C), nitrogen (N), and oxygen (O), which are derived from the DMAA-DAM monomers. The elemental composition and percentage of each element in the gel are listed in Table 1. The presence of carbon, nitrogen, and oxygen in the gel indicates that the gel contains amide- and ester-functional groups. The amide- and ester-functional groups are also confirmed via Fourier transform infrared (FTIR) spectroscopy.

### 2.9. X-ray Diffraction

**Figure 10:**
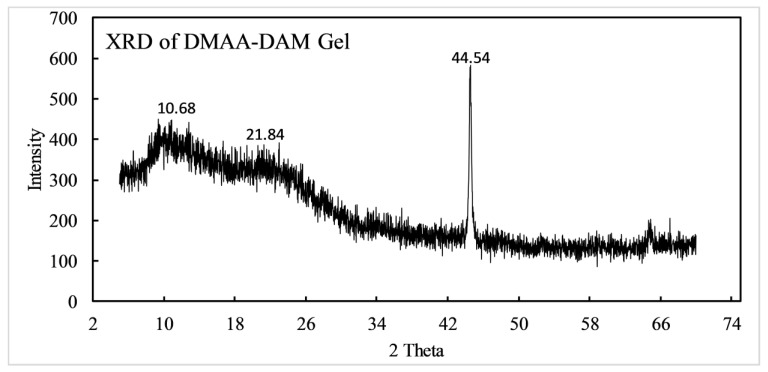
XRD peaks of DMAA-DAM gel prepared with 10 kGy radiation dose.

The XRD pattern was measured for predicting the crystallinity of the DMAA-DAM transparent gel prepared by applying a 10 kGy radiation dose, as shown in Figure 10. Shahid Bashir et al. and Hue Yang et al. investigated the XRD patterns of DMAA and poly(DMAA) where they did not find crystallinity [30,31]. In the current study, the gel was formed by crosslinking DMAA and DAM monomers using gamma irradiation. The transparency of the gel indicates that it has a homogeneous and uniform structure. However, the XRD pattern reveals some interesting features. The peak at 44.54 is for the metal plate reference and can be ignored. The gel does not give any sharp peak, indicating the absence of pure crystallinity in the structure. This means that the gel does not have a long-range order of atoms or molecules. However, there are two broad peaks at the 10.68 and 21.84 positions. The semi-crystallinity may be due to the intermolecular and intramolecular hydrogen bonding [32]. Those two peaks can be attributed to the semi-crystalline gab layer and crystalline size, respectively. Therefore, the gel can be categorized as semi-crystalline matter, which has both amorphous and crystalline regions in its structure.

## 3. Conclusions

In this work, a non-aqueous mixed solution of *N*,*N*-Dimethyla acrymamide-Diallyl Maleate (DMAA-DAM) was turned into gel by applying gamma radiation ranging from 5 to 30 kGy doses. The gel content analysis results show that all of the radiation doses produce almost the same gel products, which means that the cross-linking efficiency is not significantly affected by the dose. The equilibrium swelling decreases with the increase in the radiation dose and maximum result was found to be 1800% of dried gel prepared by applying 5 kGy. The copolymerization between two different monomers—DMAA and DAM—was analyzed and confirmed using FTIR and NMR spectroscopy. Thermal stability indicates the ability of the gel to withstand high temperatures without decomposition or degradation. The most essential characteristic of gel–thermal stability was confirmed via DSC and TGA analysis. The SEM indicated the surface morphology of the gel network where the drug can be loaded and resealed. The EDS peaks were estimated and provided the essential constituent elements with their stoichiometric percentages. XRD pattern confirmed the semi-crystalline nature of the gel. Therefore, it can be concluded that the DMAA-DAM gel can be used for drug delivery.

## 4. Materials and Methods

### 4.1. Materials and Reagents

2,3-dimethyl acrylamide and Diallyl Maleate were purchased from Merch, Germany. All samples were prepared using ultra-pure water and the temperature was kept at 198 K for the experiments. Different pH solutions were prepared by using nitric acid (HNO_3_) and ammonium hydroxide (NH_4_OH).

### 4.2. Apparatus and Instruments for the Characterization of the Gels

Different functional groups of DMAA-DAM gel were confirmed with FTIR spectroscopy (Thermo Scientific Nicolet iS50R FT-IR, Seoul, Republic of Korea). The environment of photons in the chain of the polymer was investigated using nuclear magnetic resonance (NMR) spectroscopy (JMTC-500/54/JJ, Tokyo, Japan). Thermal analyzes were performed by using differential scanning calorimetry (DSC Q100, Eschborn, Germany) and thermogravimetric analysis (TGA 8000, PerkinElmer, Waltham, MA, USA). The surface nature of the dried gel was analyzed via scanning electron microscopic (SEM) image and the constitutional elements were confirmed from energy-dispersive X-ray spectroscopic (EDS) analysis (JEOL, JSM-7900F, Tokyo, Japan). The crystallinity was checked using XRD (Smartlab, Rigaku, Tokyo, Japan).

### 4.3. Preparation of DMAA-DAM Gels with Gamma Radiation

The mixed solution of (10%) DMAA and (10%) DAM was prepared by pouring two monomers in DMSO solvent kept in 3 necked flasks followed by stirring at room temperature. Then, the sample solution was taken in 5 glass tubes, purged with nitrogen gas to remove the air from the system, and sealed for further processing. The sample tubes were kept in front of the Co-60 gamma source by maintaining a definite time and distance to adjust the radiation dose listed in Table 2. The radiation dose of this point source depends on the irradiation time and distance between a sample and the gamma source [19,33]. This table presents the changes in total effective dose (2 to 30 kGy) with the varying irradiation time and distance. Transparent gel products were obtained for all of the radiation doses except 2 kGy. After polymerization, the gel samples were carefully collected from the gamma source. In this case, the source was stopped properly and the emission was checked using dosimeter. The gels were allowed to dry inside the glass tube and were taken out once completely dry and left for further processing.

### 4.4. Extraction and Measurement of Gel Content

Despite the hydrogels being prepared in DMSO (water-soluble solvent), they were extracted in ultra-pure water. For extraction, the weight of dried hydrogel samples was measured and kept in ultra-pure water at 40 °C temperature for about 24 h to allow the contaminants and unreacted monomers and homopolymers to move out of the gel network. After complete swelling, the samples were taken out, dried in an oven at a temperature of 50 °C to remove water, and weighed. Finally, the gel content was calculated by using the following equation [34]:

(1) $$Gel\;fraction\left[\%\right]=\frac{W_{1}}{W_{0}}\times 100$$

where *W*_0_ and *W*_1_ are the dried gel weights before extraction and after extraction, respectively.

### 4.5. Measurement of Hydrophilicity/Equilibrium Swelling

The equilibrium swelling was also performed in a neutral pH solution (ultra-pure water) at room temperature. The extracted dried gel samples were soaked in a neutral solution for 12 h followed by measuring weight until the constant weight reached (up to 14 h). From the dried and swelled weight, the equilibrium swelling was evaluated by using the following equation [35]:

(2) $$Water\;absorption\left[\%\right]=\frac{W_{t}-W_{1}}{W_{1}}\times 100$$

where *W*_1_ and *W_t_* are the dried gel weight and the gel weight after swelling in the solution, respectively. The swelling experiments were performed three times to check the accuracy.

### 4.6. Characterization of Gel

All of the characterizations were performed for the prepared gel by applying a 10 kGy gamma radiation dose.

#### 4.6.1. Characterization with FTIR Spectroscopy

Fourier transforms infrared spectroscopic analysis has two types of vibrational modes—stretching and bending—which happen very quickly (one cycle takes 10–15 s) during the measurement. The reference KBr was used with the range from 700 cm^−1^ to 4000 cm^−1^.

#### 4.6.2. Characterization with Proton NMR Spectroscopy

^1^H Nuclear magnetic resonance spectroscopic analysis was performed to indicate the environment of the protons in the DMAA-DAM gel by dissolving it in chloroform solvent. The chemical shift range was maintained at 0 to 8 ppm.

#### 4.6.3. Thermal Analysis with DSC and TGA

Differential scanning calorimetry of the gel was carried out under a continuous 50 mL/minute N_2_ flow with a heating rate of 10 °C/min and with thermogravimetric analysis under a 25 mL/minute N_2_ gas flow with a heating rate of 10 °C/min. Thermal analysis DSC was carried out over a temperature range of 25 °C to 300 °C to investigate the initial changes upon heating the gel sample. In this study, the TGA curve of the gel was obtained by heating the sample from 25 °C to 600 °C at a constant rate and recording the weight loss at different temperatures.

#### 4.6.4. Surface Analysis with SEM-EDS

The SEM-EDS analysis requires the sample to be coated with a conductive material, such as platinum, to prevent charging and improve image quality. In this study, the SEM-EDS analysis was performed on the dried DMAA-DAM gel with 5 nm platinum coating.

#### 4.6.5. Characterization with X-ray Diffraction

The X-ray diffraction pattern was measured for predicting whether the gel is crystalline or amorphous. The XRD was run over a range of 5 °C to 70 °C with a scene rate of 2 °C/min, and a metal reference plate was used.

## Figures and Tables

**Table 1:** Significant constitutional elements of DMAA-DAM.

Element	Weight%	Weight% Sigma
Carbon (C)	66.37	0.42
Nitrogen (N)	14.12	0.50
Oxygen (O)	19.51	0.22
Total	100.0	-

**Table 2:** Radiation dose and respective gel products for DMAA (10%) and DAM (10%).

Radiation Dose (kGy)	2	5	10	20	30
Dose rate (Gy/hr)	86	217	437	823	1310
Distance from the source (cm)	46	27	17	13	8
Irradiation time (hr)	24	24	24	24	24
Gel product	No gel	Solid gel	Solid gel	Solid gel	Solid gel

## Data Availability

BHUYAN, MD (2023), “Dimethyl acrylamide-Diallyl Maleate gel/hydrogel in Non-aqueous solution and characterization”, Mendeley Data, v1http://dx.doi.org/10.17632/cbvkm5dtk4.1. 28 June 2023.
